# Impingement-free hip range of motion after osteochondroplasty and relative neck lengthening in adults with healed Perthes disease

**DOI:** 10.1186/s13018-020-01899-w

**Published:** 2020-08-26

**Authors:** Mohammed Elmarghany, Tarek M. Abd El-Ghaffar, Ahmed Elgeushy, Yehia Hasanin, Ehab Elzahed, Mohamed I. Abulsoud, Mohamed Moawad

**Affiliations:** Orthopedic Department, Faculty of Medicine, Al-Azhar University Hospitals, Cairo, 11675 Egypt

**Keywords:** Legg-Calvé-Perthes disease, Osteochondroplasty, Safe hip dislocation

## Abstract

**Objective:**

Our main objective is to assess the efficiency of the osteochondroplasty with relative neck lengthening in adults with healed Perthes clinically (through assessment of impingement-free hip ROM, functional scores) and radiographically.

**Patients and methods:**

This was a prospective case series study included 30 hips of 30 patients who underwent osteochondroplasty and RNL due to symptomatic healed LCPD. This study included 16 males (53.3%) and 14 females (46.7%). The age of patients ranged from 19 to 40 years with mean age 26.4 years at the date of surgery (SD 6.4).

**Results:**

Median time of follow-up was 27.7 months after surgery (range 12–60 months). Two patients (6.6%) developed avascular necrosis (AVN) and needing total hip replacement; none of our patients developed nerve injury, detachment of the trochanteric fragment, and wound infection needing treatment. Preoperative Stulberg classes II and III improved more than preoperative Stulberg classes IV and V, although not statistically significant (*P* = 0.1104, *n* = 30). The mean HHS and WOMAC score values for each patient were higher in the Stulberg II and III groups compared to the Stulberg IV and V groups

**Conclusion:**

Head and neck osteochondroplasty performed through the surgical dislocation approach, combined with RFNL, relieved pain and restored function in most of the patients with reasonable complications.

**Level of evidence:**

IV

## Introduction

The hip with healed Legg-Calvé-Perthes disease (LCPD) usually have one or more of these abnormal pathoanatomies: dysplastic acetabulum; abnormal shape of femoral head (large, cone -shaped, flat, mushroom); short, thickened and varus femoral neck; and over-riding greater trochanter (functional coxa vara) [1].

The etiology of LCPD is still debated as the literature available shows major limitations in terms of great heterogeneity and a lack of high-profile studies. In the systematic review done by Pavone et al. [2] which include 64 articles, they reported that in the role of environmental risk factors genetic factors, a congenital or acquired predisposition cannot be excluded in disease pathogenesis. One of the most supported theories involved mechanical induced ischemia that evolved into avascular necrosis of the femoral head in sensible patients [2].

The most used classification that determines the degree of deformity of LCPD hip is the Stulberg classification which correlates with the long-term outcome. Class I and II (spherical congruency) conveys the best prognosis and class III and IV (aspherical congruency) a little worse prognosis. Class V (aspherical incongruency) carries the worst prognosis and more risk of developing osteoarthritis (OA) at a younger age [1].

The deformity resulting from LCPD including a high-riding greater trochanter, a short femoral neck, and aspherical femoral head-neck junction causes femoroacetabular impingement (FAI) and/or instability of the hip which may finally cause damage and subsequently OA of the hip resulting in hip pain, restricted range of motion, and impaired abductor function [3–5].

The morphology of the proximal femur after healed Perthes disease is the single most important factor predicting the long-term outcome [5].

The main goal in the management of post-Perthes sequelae is to improve hip mechanics (eliminate femoroacetabular impingement and improve abductor lever arm), relieve pain, improve hip motion, improve hip function for daily living, and enhance the quality of life and physical activity level. This is may be done by reshaping the femoral head-neck junction, increasing the length of femoral neck, and advancement of greater trochanter thus improving the intraarticular and extraarticular cause of impingement [6].

These aims can be achieved through surgical dislocation of the hip (SHD), described by professor Ganz in 2001 which can help us to address all proximal femoral pathoanatomy without risk on blood supply of femoral head [7, 8].

In healed LCPD, the main procedures used to correct proximal femoral pathoanatomy are osteochondroplasty and relative femoral neck lengthening. Osteochondroplasty reshapes the femoral head, while relative neck lengthening (RNL) is achieved by distal advancement of the greater trochanter and subperiosteal trimming of the proximal stable trochanter. This improves abductors function by increasing their lever arm [7, 8].

## Material and methods

From March 2015 to March 2020, a prospective case series study was done at Al-Azhar University hospital (Al-Hussein Hospital), Cairo, Egypt, on patients with healed LCPD treated with osteochondroplasty and RNL using surgical hip dislocation (SHD) approach. Patients with severe hip OA, age > 50 years were excluded from this study.

This study included 30 hips of 30 patients who underwent osteochondroplasty and RNL due to symptomatic healed LCPD. This study included 16 males (53.3%) and 14 females (46.7%), The age of patients ranged from 19 to 40 years with mean age was 26.4 years at the date of surgery (SD was 6.4).

Rt hip was affected in 16 patients (53.3%) and Lt hip in 14 patients (46.7%); 17 patients were Stulberg class II,III, whereas 13 patients were Stulberg IV,V. All of our patients have positive anterior impingement test. The median time of follow-up was 27.7 months after surgery (range 12–60 months) (Table 1).

**Table 1:** Demographic criteria of our patients

Patient number	Age	Sex	Side	Max follow-up (months)	Stulberg class	Anterior impingement test
1	19	M	Rt	60	III	+ve
2	23	F	Rt	60	IV	
3	25	F	Lt	56	II	
4	30	M	Lt	53	V	
5	26	M	Lt	50	II	
6	22	F	Rt	50	III	
7	20	M	Rt	49	IV	
8	20	M	Lt	40	III	
9	28	F	Lt	30	IV	
10	28	M	Lt	30	III	
11	33	F	Rt	30	IV	
12	38	M	Rt	30	III	
13	40	F	Rt	29	IV	
14	37	F	Rt	26	II	
15	30	M	Lt	25	II	
16	32	F	Rt	25	II	
17	30	F	Rt	23	II	
18	20	M	Rt	22	II	
19	19	M	Rt	22	IV	
20	20	F	Rt	20	II	
21	19	M	Lt	20	III	
22	22	F	Rt	18	III	
23	21	M	Rt	16	IV	
24	34	M	Lt	15	III	
25	29	F	Lt	15	IV	
26	26	M	Rt	14	IV	
27	33	F	Lt	14	IV	
28	23	M	Lt	13	IV	
29	19	F	Lt	12	IV	
30	20	M	Lt	12	III	

All patients were evaluated clinically and radiologically. Clinical examination included hip range of motion (especially flexion, abduction, and internal rotation), leg-length discrepancy (LLD), and presence of Trendelenburg sign or limp, hip extensor, and hip abductor strength. Range of motion was measured using a goniometer.

Strength of hip abductors and extensors were estimated using a 5-graded scale: “0 no contraction, 1 flicker or trace of contraction, 2 active movement, with gravity eliminated, 3 active movement against gravity, 4 active movement against gravity and resistance, 5 normal power.”

**Fig. 1:**
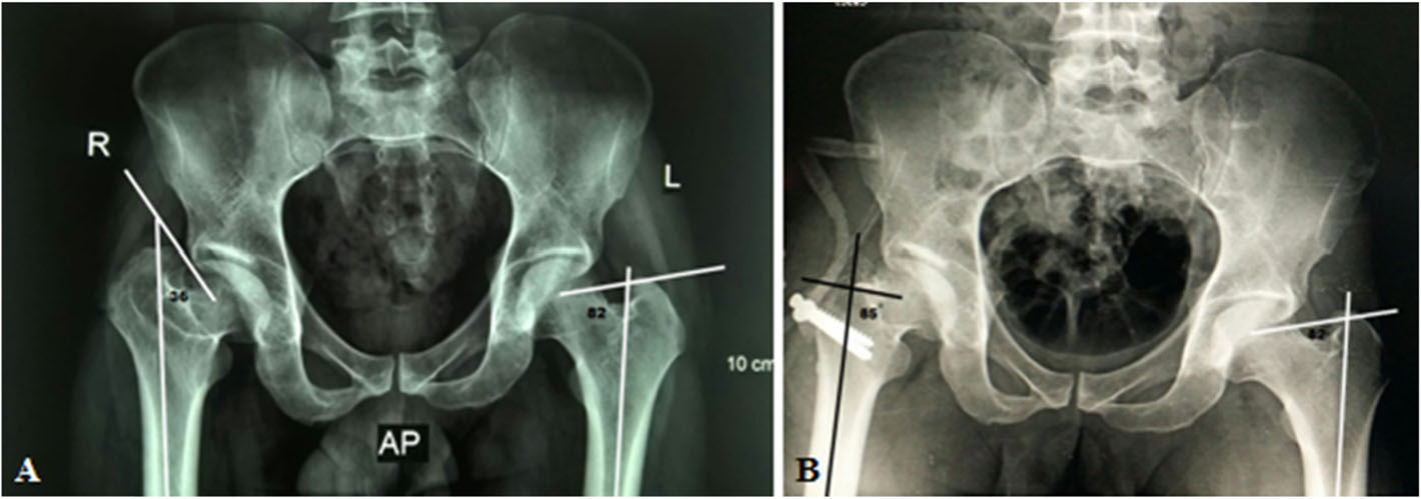
Anatomical proximal femoral angle preoperative (**a**) and postoperative (**b**) in patient no. 10 (from center of the head to the tip of greater trochanter)

**Fig. 2:**
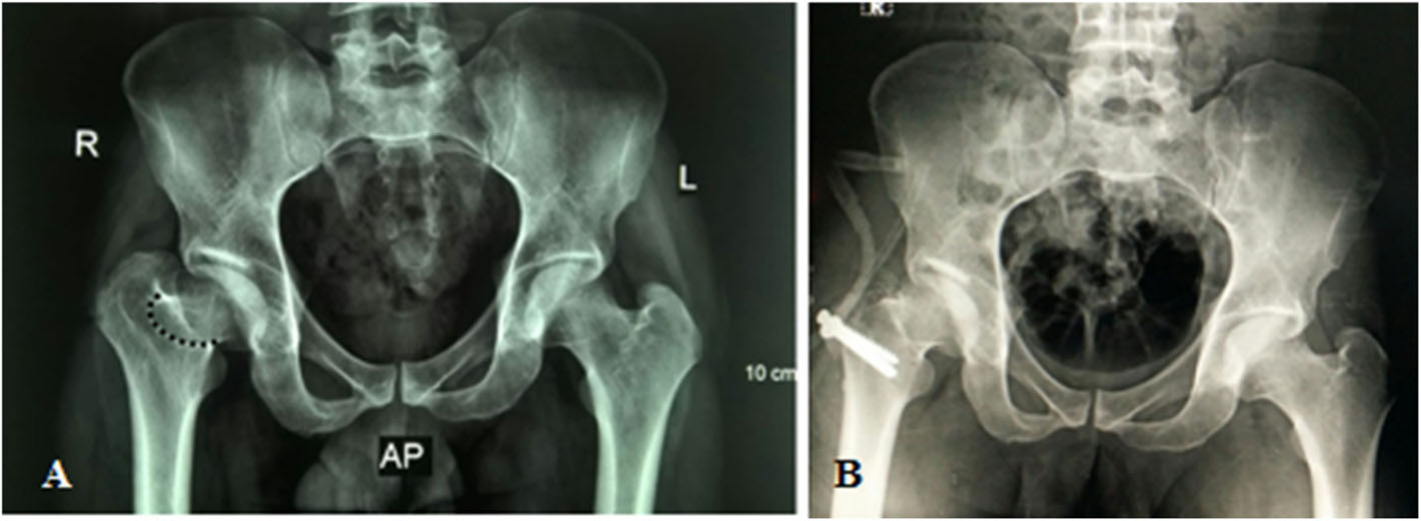
Sagging rope sign in patient no. 10 present preoperative (**a**) and absent postoperative (**b**); this means the anteroinferior projection of FH was removed

**Fig. 3:**
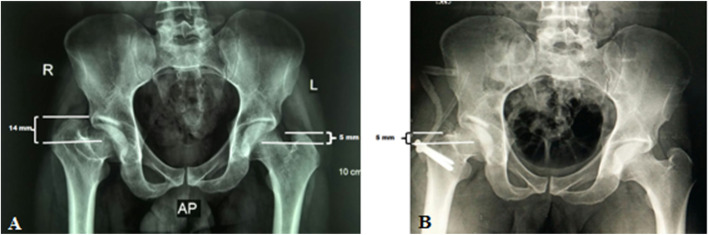
Center-trochanteric distance (CTD) preoperative (**a**) and postoperative (**b**) in patient no. 10

**Fig. 4:**
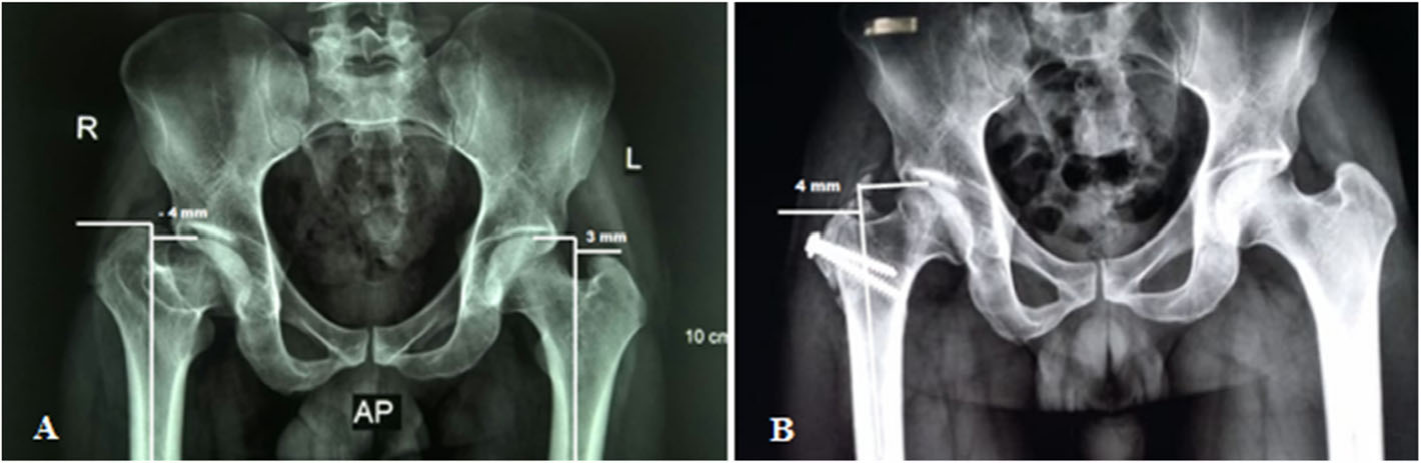
Articulo-trochanteric distance (ATD) preoperative (**a**) and postoperative (**b**) in patient no. 10

**Fig. 5:**
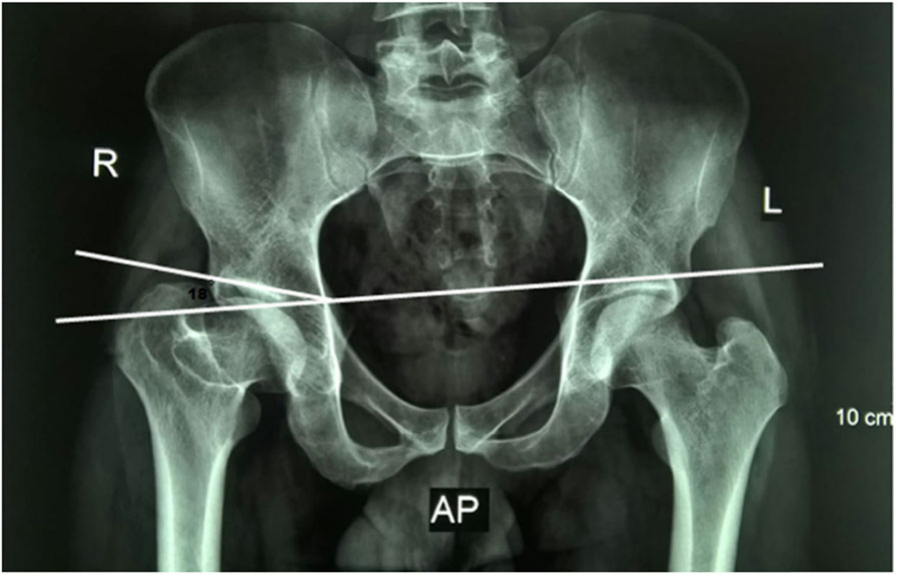
Actabular angle of Tonnis in patient no. 10 (horizontal line is parallel to the inter-tear drop line and the other line from the medial end of the sourcil to the lateral end of the sourcil)

Radiological evaluation of our patients based on preoperative and postoperative anteroposterior (AP) pelvis radiographs. The Stulberg classification was used to examine the sphericity of the hip joint. Anatomical proximal femoral angle was used to evaluate functional coxa vara (Fig. 1). Anteroinferior femoral head protrusion (sagging rope sign) can be visualized in preoperative anteroposterior radiographs and was absent in postoperative ones (Fig. 2). The center-trochanteric distance (CTD) was used to estimate trochanteric overgrowth (Fig. 3). Articulo-trochanteric distance (ATD) was used to evaluate abductor length and efficiency (Fig. 4). Acetabular angle of tonnis was used to evaluate acetabular dysplasia (Fig. 5).

### Surgical technique

**Fig. 6:**
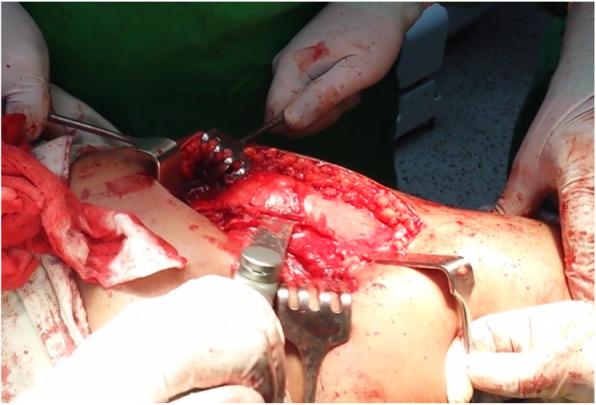
Trochanteric osteotomy of patient no. 10

**Fig. 7:**
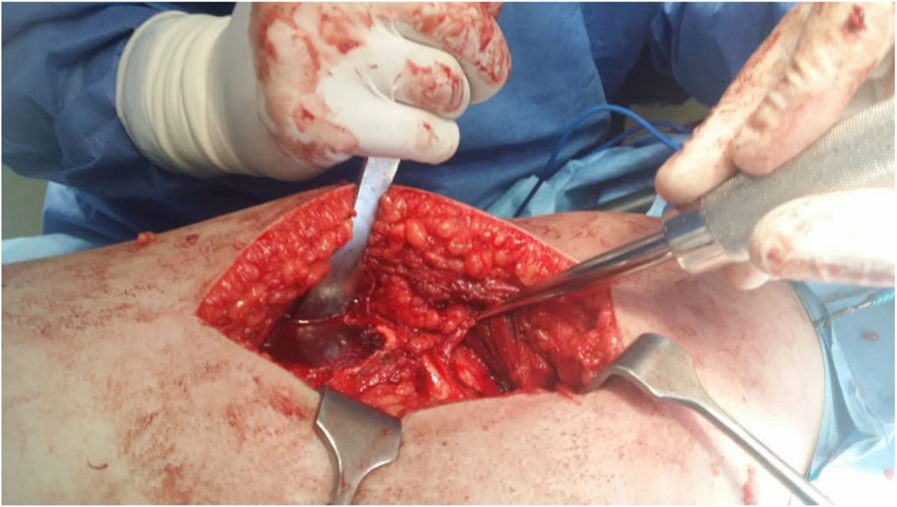
Interval between gluteus minimus and piriformis in patient no. 20

**Fig. 8:**
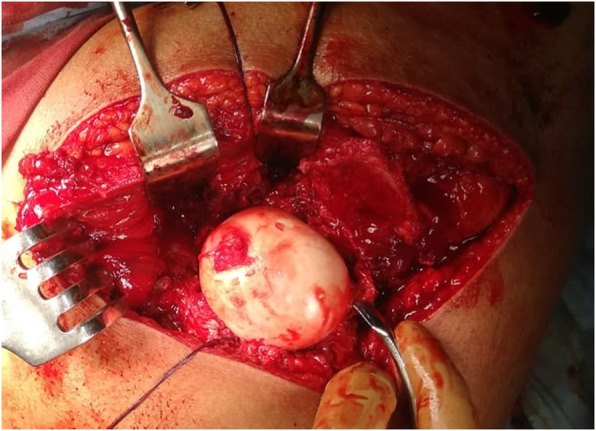
The configuration of the femoral head in patient no. 20

**Fig. 9:**
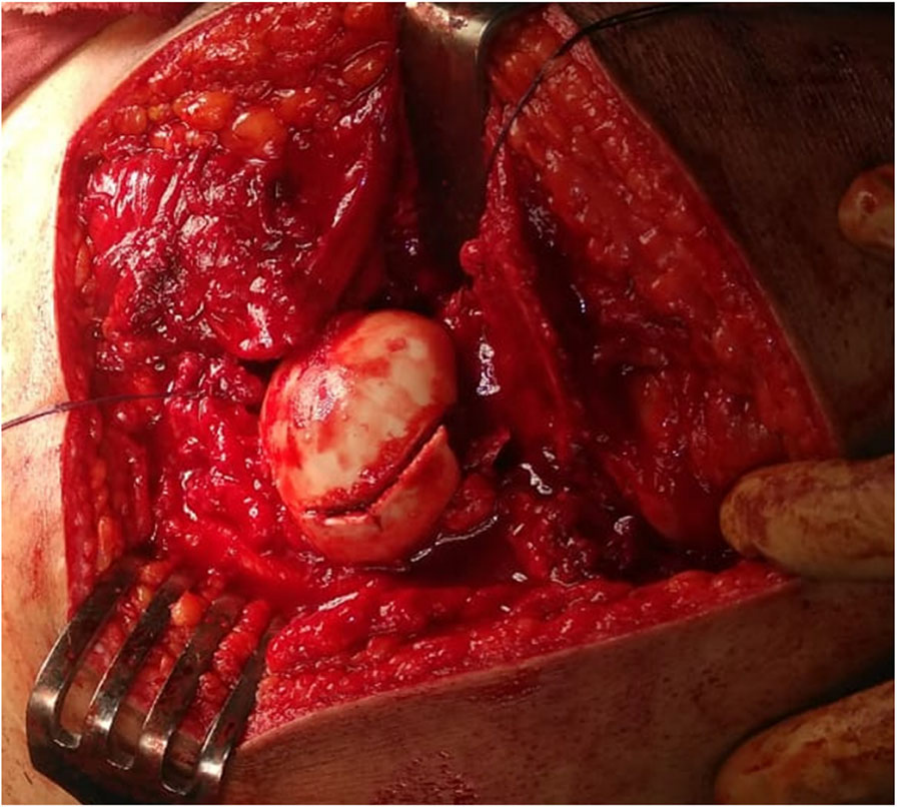
Osteochondroplasty in patient no. 10

**Fig. 10:**
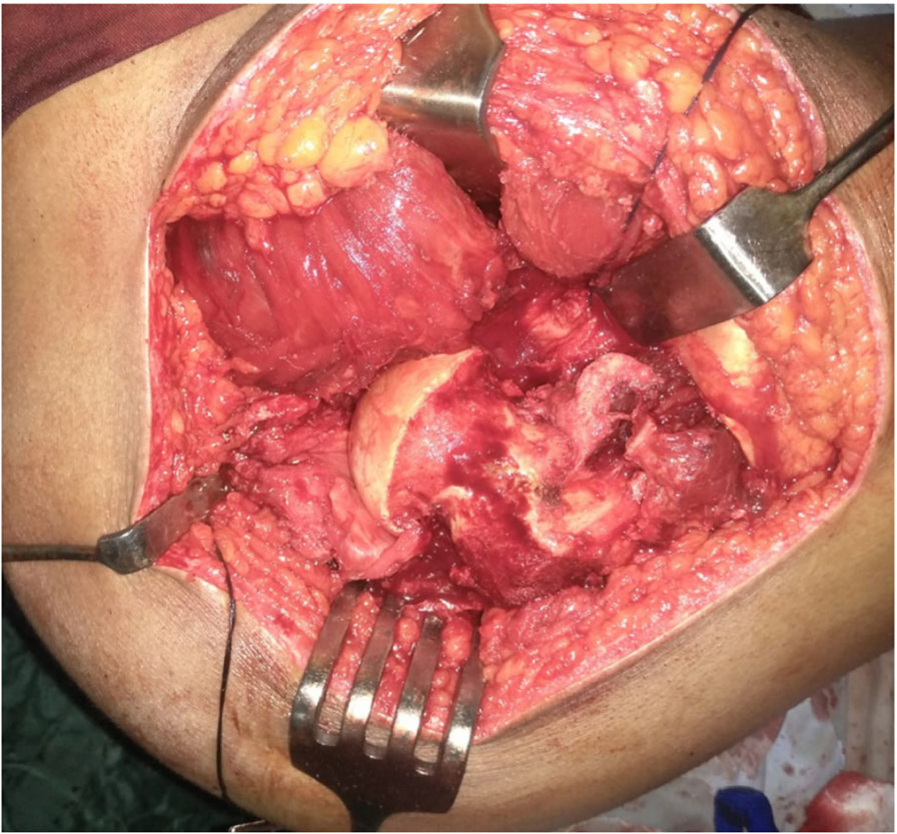
The femoral head became more spherical and the femoral neck more long after reshaping of the proximal femur in patient no. 10

- The patient was placed in a lateral decubitus position.
- A straight lateral incision was made centered over the greater trochanter.
- The approach was through the Gibson interval between tensor fascia lata and gluteus maximus.
- Trigastric trochanteric osteotomy (Fig. 6)
- Interval between gluteus minimus and piriformis (Fig. 7)
- Capslutomy, Z-shaped, or reversed Z-shaped
- Dislocation of the hip after cutting of ligamentum teres (Fig. 8)
- Examination of the acetabulum for any chondral and labral injury
- Reduction of the hip again and creation of the extended retinacular flap(anteroinferior flap and posterosuperior flap)
- Redislocation of the hip and reshaping of the head and neck and creation of clear head-neck offset (Figs. 9 and 10)

### Statistics

Data were coded and entered using the statistical demo version of the Graph Pad InStat 3. Continuous variables are presented as mean ± SD, and qualitative variables as percentages. Qualitative variables were compared among groups using the Chi-square test (*χ*^2^) test, whereas continuous variables were compared with the analysis of variance test. *P* value < 0.05 was considered statistically significant whereas *P* value > 0.05 was considered statistically non-significant.

## Results

Median time of follow-up was 27.7 months after surgery (range 12– 60 months). Two patients (6.6%) developed avascular necrosis (AVN) and did total hip replacement; none of our patients developed nerve injury, detachment of the trochanteric fragment, and wound infection needing treatment. Preoperative Stulberg classes II and III improved more than preoperative Stulberg classes IV and V, although not statistically significant (*P* = 0.1104, *n* = 30). The mean Harris Hip score (HHS) and WOMAC score values for each patient were higher in the Stulberg II and III groups compared to the Stulberg IV and V groups (Table 2).

**Table 2:** Postoperative clinical scores in different Stulberg groups

	Stulberg group	no.	Mean	SD	SD error mean	Paired *t* test (*P* value)
HHS score	II, III	17	93.4	10	2.4	0.1104
	IV, V	13	84.2	12.3	3.4	
WOMAC score	II, III	17	4	2.3	0.56	0.2983
	IV, V	13	5.5	2.5	0.69	

*HHS* Harris Hip score, *WOMAC* Western Ontario and McMaster Universities Osteoarthritis Index

Mean postoperative flexion range of motion (ROM) was 116.9 (SD was 11). In comparison with preoperative ROM (50.7 ± 9.7) and internal rotation with hip flexion and abduction range improved postoperative as shown in Table 2 (*P* value < 0.0001, considered extremely significant) (Table 3).

**Table 3:** Comparison of pre- and postoperative hip ROM

ROM	Preoperative	Postoperative	Paired *t* test
	Mean ± SD	Mean ± SD	*P* value (significance)
Flexion	50.7 ± 9.4	116.9 ± 11	< 0.0001 (ES)
IR with flexion	7.75 ± 3.7	42.6 ± 7	
Abduction	23.4 ± 5.8	44 ± 2.8	

*IR* Internal rotation, *SD* Standard deviation, *ES* Extremely significant*, ROM* Range of motion

Mean postoperative HHS was 89.4 (ranged from 65 to 100) (SD was 12.8). The improvement of clinical score was significant. Mean improvement in HHS was 22.9 (SD 8.6) (*P* value < 0.0001, considered extremely significant) (Table 4).

**Table 4:** Comparison of pre- and postoperative HHS

HHS				Paired *t* test
	Range	Mean ± SD	Improvement mean ± SD	*P* value (significance)
Preoperative	60–73	66.5 ± 3.9	22.9 ± 8.6	< 0.0001 (ES)
Postoperative	65–100	89.4 ± 11.8		

*SD* Standard deviation, *ES* Extremely significant

Postoperative WOMAC score was used to assess 30 patients, 6 months postoperatively and full union of osteotomy side and regaining of functional activities. Mean postoperative WOMAC score was 4.6 (ranged from 0 to 11) (SD was 2.5). In comparison with preoperative, mean improvement were 85.12 (SD 4.7), (*P* value < 0.0001, considered extremely significant) (Table 5).

**Table 5:** Comparison of pre- and postoperative WOMAC score

WOMAC score				Paired *t* test
	Range	Mean ± SD	Improvement mean ± SD	*P* value (significance)
Preoperative	80–94	88.4 ± 3.8	85.12 ± 4.7	< 0.0001 (E S)
Postoperative	0–11	4.6 ± 2.5		

*SD* Standard deviation, *ES* Extremely significant

**Fig. 11:**
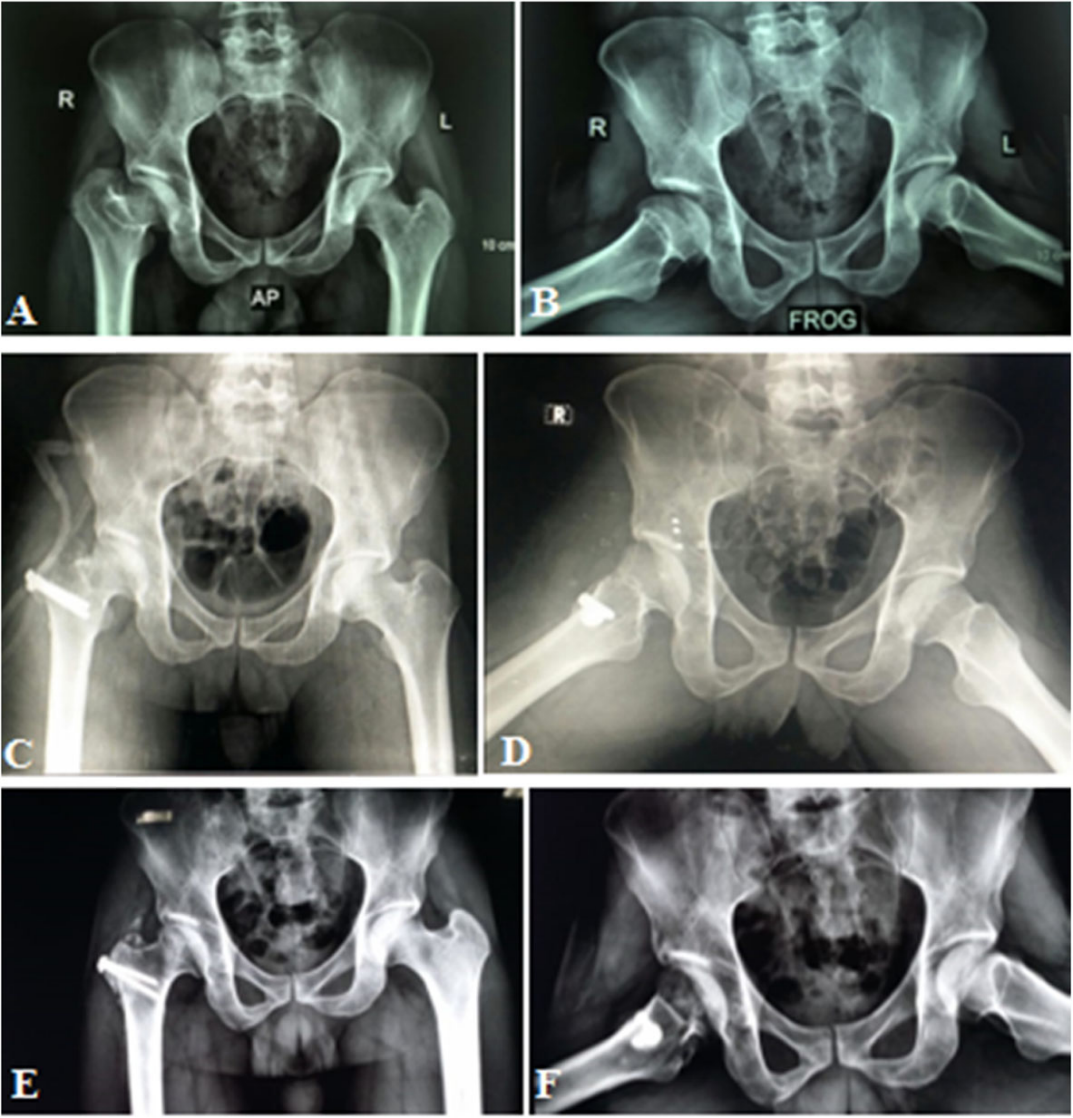
Radiographic evaluation of patient no. 10, preoperative (**a**, **b**), immediate postoperative (**c**, **d**), and 15 months postoperative (**e**, **f**)

**Fig. 12:**
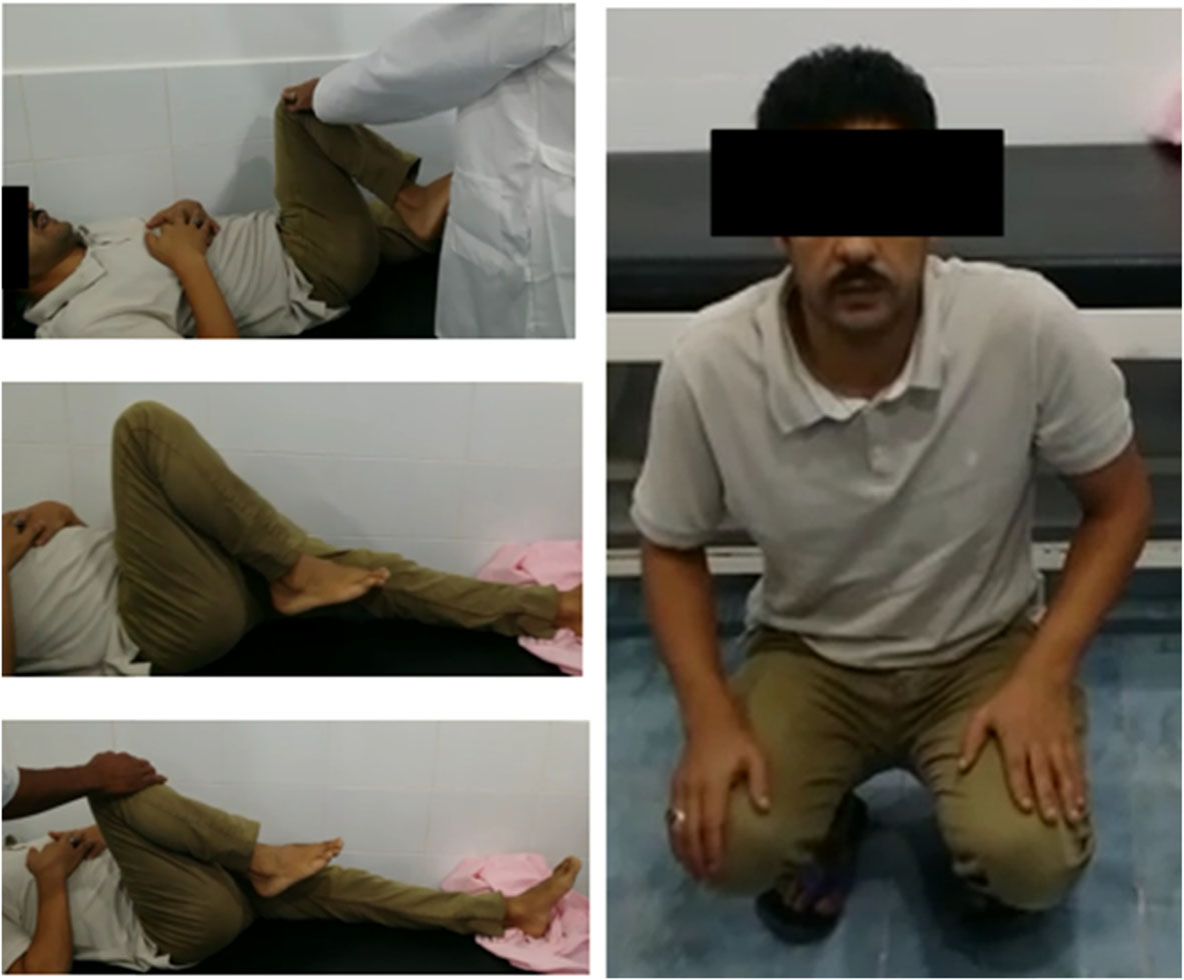
Clinical evaluation of 15 months postoperative of patient no 10

Typical clinical and radiologic appearance after relative femoral neck lengthening and head-neck osteochondroplasty showed significant improvement; however, no periacetabular osteotomy was done for acetabular dysplasia (Table 6, Figs. 11 and 12).

**Table 6:** Comparison of pre &postoperative radiographic appearance

Radiological evaluation					Paired *t* test
	Anatomical mPFA	CTD	ATD	AA of Tonnis	*P* Value
Preoperative	50.4° ± 9.3	15.6 mm ± 2	− 5.47 mm ± 1.8	16° ± 2.7	< 0.0001 (ES)
Postoperative	87.7° ± 1.9	15.6 mm ± 2	5.5 mm ± 0.8	-----	

*mPFA* Medial proximal femoral angle, *CTD* Centero-trochanteric distance, *ATD* Articulo-trochanteric distance, *AA* Acetabular angle, *ES* Extremely significant

## Discussion

The majority of patients showed remarkable improvement (94%). Preoperative Stulberg II and III patients showed better results than Stulberg IV and V patients (Stulberg II and III improvement in 17 patients (56.6%), Stulberg IV and V 13 patients (44.4%), *n* = 30, *p* = 0.1104).

Clohisy et al. [9] reported good results for 6 patients treated with SHD and periacetabular osteotomy (PAO), as did Anderson et al. [8] for a group of 14 patients treated with SHD and trochanteric advancement of which 1 had staged PAO performed.

Good results were also reported by Albers et al. [10] for a more heterogeneous patient group (53 patients of which 40 patients were treated with SHD and 57% by additional previous surgery), as have Shore et al. [11] for a group of 29 patients treated with SHD and head-neck osteochondroplasty of which 26 patients had needed additional procedures.

Risto et al. [12] for homogenous group of patients, 12% of the patients in their study had gone through THR. More Stulberg II and III patients reported improvement after surgery than Stulberg IV and V patients, but there were no statistical differences between the groups regarding self-assessed score values.

In this study, we conducted the technique of SHD to do osteochondroplasty and relative neck lengthening without PAO to address intraarticular FAI and abduction mechanics for homogenous group of patients with the same diagnosis. By the time of follow-up, 6.6% of the patients in this study had gone through THR. More Stulberg II and III patients reported improvement after surgery than Stulberg IV and V patients, but there were no statistical differences between the groups although the number of Stulberg IV and V patients was high (46%). Anderson et al. [9] reported failures for three of nine patients with preoperative Stulberg class IV. Albers et al. [10] also claims that preoperative Stulberg class is prognostic for “midterm results”. One explanation of this effect could be that the surgery improves range of motion and mechanical properties for hips with a preexisting congruency for the Stulberg II and III patients which the Stulberg IV and V patients are lacking. The 2 patients who had a total hip replacement were Stulberg class IV. HHS had been improved from mean 66.5 preoperatively to 89.4 postoperatively (*p* < 0.0001) and WOMAC score had been improved from mean 84.4 preoperatively to 4.6 postoperatively (*p* < 0.0001).

The strength points of this study represented in the following:

- Large number of patients having the same diagnosis
- Relatively long follow-up period
- Prospective nature
- Only single technique of surgery was used (no concomitant PAO was used); really in the beginning of this study, we had discussed the surgical techniques for many patients who have also acetabular dysplasia and they preferred staged surgery; then, later on, after their satisfaction, a lot of them refused to do other surgeries, so the little number of patients who did PAO were excluded from this study. This was also noticed by Risto et al. [12] whose patients with additional PAO had a worse prognosis; they still did better than those patients with acetabular dysplasia not operated on with additional PAO. Novais [13] also recommend staged PAO. Clohisy et al. [14] showed good results for addition of PAO.

The limitations of this study represented in the following:

- Short follow-up period to decide the prognostic significance of Stulberg IV and V that may need mid-term to long-term follow-up. Mid- and long-term follow-up studies are critical to demonstrate if this surgical approach provides durable pain-free hip function and avoidance of later THA.
- Lack of comparison group
- The lack of treatment of the acetabular side may predispose to potentially recurrent labral pathology and continued instability and recurrence of pain, so long-term follow-up is recommended.

## Conclusion

The surgical hip dislocation approach allows identifying sources of impingement, and treating intraarticular and extraarticular abnormalities. Head and neck osteochondroplasty performed through the surgical dislocation approach, combined with RFNL relieved pain and restored function in most patients with no major complications.

## Data Availability

All data and materials are available.

## Abbreviations

RFNL: Relative femoral neck lengthening
HHS: Harris Hip score
ROM: Range of motion
PAO: Periacetabular osteotomy
ES: Extremely significant
SHD: Surgical hip dislocation
AP: Anteroposterior
